# We must discuss research environments

**DOI:** 10.1098/rsos.231742

**Published:** 2024-06-26

**Authors:** John Gallacher, Chris Webster

**Affiliations:** ^1^ Department of Psychiatry, University of Oxford, Oxford, UK; ^2^ Healthy High Density Cities Lab, Department of Urban Planning and Design, HKU Institute of Data Science, HKU Urban Systems Institute, Hong Kong University, Pok Fu Lam, Hong Kong

**Keywords:** research environment, club theory, collaboration, data market, innovation

## Abstract

A major challenge facing the biomedical community is creating and sustaining high-quality research environments. A literature search identified five common themes underlying biomedical research environments comprising collaboration, data access, user-led innovation, data provenance and a deep commitment to public and scientific benefit. Club theory is used to develop a model describing social structures that underpin these themes. It is argued that collaboration underlies impactful science and that collaboration is hindered by high transaction costs. This, combined with poorly defined property rights surrounding publicly funded data, limits the ability of data markets to operate efficiently. Although the science community is best placed to provide solutions for these issues, incentivization by funding agencies to increase the benefits of collaboration and reduce uncoordinated activity will be an accelerator. Given the complexity of emerging datasets and the collaborations needed to exploit them, trust-by-design solutions are suggested. The underlying motivational ‘glue’ that holds this activity together is the aesthetic and ethical value base underlying good science. The model has implications for data-driven science more generally. As biomedical science in the Global South develops, there is an opportunity to address foundational structural issues prospectively rather than inherit unwanted constraints of current practice.

## 1. Introduction: context is everything

One of the highlights of being a scientist is belonging to a truly international community that transcends race and creed in making sense of the world around us [1]. The key to the effectiveness of this community is creating and sustaining high-quality research environments. However, what defines ‘high quality’ remains ambiguous. For example, in the 2021 UK Research Excellence Framework exercise [2], research environment is assessed independently of outputs and impact. Although 15% of total ranking scores are given to the research environment, this is defined non-specifically in terms of ‘vitality and sustainability’ [3]. The potential scientific, health and wealth benefits of high-quality research environments are not disputed. This is reflected in the UK Government’s roadmap to make the UK a partner of choice for research and development [4]. However, this policy statement is focused on outcomes rather than determinants; raising the question of what constitutes a research environment that will achieve ‘partnership of choice’?

For biomedical science, and particularly for research-cohort data, the question of what constitutes a high-quality research environment is acute given the complexity, breadth and depth of data and disciplines involved. Specific features of research activity, such as networking and collaboration [5], productivity [6] or impact [7], command a growing literature [8–10]. The Royal Society has identified improved peer esteem, improved culture-setting by leaders, greater career mobility, open science and fostering scientific leadership as keys to research environment quality [11]. The Wellcome Trust has identified changes in funding structures, support for early career researchers and improved training and policies as areas for improvement [12]. These proposals reflect a mixture of laboratory culture and opportunity improvement. Important as culture and opportunity are, however, they are both contingent upon a wider socio-economic context. Here, we argue that it is helpful to characterize this context and consider whether it describes a general framework that can inform the development of specific solutions for particular communities, i.e. help them shape their environment to scientific advantage.

The problem is framed in terms of science being a data-driven economy. From this perspective, we translate the question ‘what constitutes a high-quality research environment?’ into ‘what structures better promote growth in a data-driven economy?’ Implicit in this definition is how these structures incentivize participation in this economy. These narrow definitions simplify a complex problem in terms that are generalizable across disciplines and provide conceptual insights [13]. Also, growth in the size and complexity of the science economy may be considered a proxy for diverse other metrics such as opportunity, culture, career progression and outputs.

We use club theory as a conceptual model to provide a conceptual framework for understanding how socio-economic relationships relevant to biomedical research environments work out in practice [14]. In club theory, a club is a group of people sharing a public good. A public good is a good or service that, when not congested, is perfectly jointly consumed in the sense that one person’s consumption does not diminish the consumption of another. Organizing such goods in clubs can help keep them uncongested. Clubs can be formal enduring organizations or less-formal project-specific collaborations. Here, we use club in this latter sense of collaboration around a research theme or question. Club theory has been applied in a variety of behavioural and organizational contests [15,16]. We use club theory to understand how scientists self-organize to develop the institutions and structures that characterize research environments. By ‘institutions’, we mean informal and formal systems of rules and sanctions that are used to guide practice. By ‘structures’, we mean the stable arrangement of institutions that govern community behaviour: scientific rigour is an institution, whereas peer review is a structure. In this context, ‘organizations’ refer to formal groups, such as research institutes, working together to create complex knowledge.

Within a club-theoretic framework, institutions and structures emerge to reduce the cost and increase the benefits of voluntary working together. Decisions are made by individuals regarding the value (personal and/or social) of joining the club (joining a collaboration), and by club members on the value of extending membership (increasing the number of collaborators). In this model, the benefit of club membership is the ability to address specific scientific questions with greater precision at lower cost. The value of club membership is dynamic and varies according to the state of knowledge, the development of technology, etc. Although clubs ‘come and go’, the institutions, structures and organizations upon which they rely are less agile. They require revision if they are not, over time, to lose efficiency in supporting the creation of complex knowledge.

We characterize the biomedical research environment by means of a literature review and then use club theory to examine the underlying structures that affect how these characteristics are expressed. We focus on collaboration, data access, innovation, trust and motivation, and use these themes to propose a general approach rather than specific solutions.

## 2. Methods

To characterize the biomedical research environment, we conducted a search of Medline, Embase and PsycINFO using Ovid [17]. The search term was ‘research environment [Title]’. The census date was 31 December 2023. Inclusion criteria were publications in peer-reviewed journals, in the English language, full text freely available and referring to human environments. The full text was used to characterize articles according to the main themes addressed. This was assessed subjectively and simple counts were used; the articles were not weighted for importance.

## 3. Results

### 3.1. Search results

The search strategy generated 170 peer-reviewed English language publications, of which the full text was available for 162, of which 158 referred to human environments. The articles comprised research papers, editorials, commentaries, conference abstracts and monographs. The list of articles and their categorization can be found in the electronic supplementary material.

### 3.2. Recurring themes

Of the 158 eligible publications, 44 referred to the research environment in general and the importance of a supportive research infrastructure. Five specific recurring and overlapping themes emerged comprising collaboration (*n* = 31 (20%)), data-related technologies (*n* = 65 (41%)), innovation (*n* = 47 (30%)), data provenance and governance (*n* = 23 (15%)) and motivation (including mental health) (*n* = 26 (17%)), with many studies covering more than one theme. Most overlap was found between the data-related technologies and innovation (table 1). Of the 64 and 47 studies focusing on data-related technologies and innovation, respectively, 41 addressed both themes. These themes may be considered indicative rather than definitive. Nevertheless, they identify characteristics of the research environment around which biomedical scientists self-organize and can be used as a basis for a model of the biomedical research environment.

**Table 1.** Distribution of research environment themes across 158 full-text English language publications (individual publications may refer to more than one theme).

frequency (%)				
collaboration	data-related technologies	innovation	data provenance	motivation
31 (20%)	65 (41%)	47 (30%)	23 (15%)	26 (17%)

## 4. Model development

We synthesize these data from the perspective of a scientific collaboration or ‘club’. We frame these themes to accommodate an analysis of social structures that impact the operation of a data-driven economy; considering the collaborative imperative, effective data markets, user-led innovation, trust-by-design and the foundations of scientific motivation.

### 4.1. The collaborative imperative

Science illustrates the compelling advantage of collaboration. Every further step in specialization is a step towards greater interdependence between scientists in addressing emerging questions. However, limited opportunity (or scarcity of reward), such as achieving promotion or a high-impact publication, drives competition. The extent to which competition *per se* has generated insights that would not otherwise have been made is unknowable. In contrast, the extent to which collaboration has generated insights that would not otherwise be made is easily measured by the author list on any peer-reviewed publication. These lists measure the revealed preference for collaboration over exclusivity in creating complex knowledge. A club-theoretic approach suggests scientists assess the value of joining or forming a collaboration (club) according to the net benefits associated with membership. Similarly, existing collaborators (club members) assess the value of extending membership based on its net benefits. In this model, net benefit of collaboration is the ability to address specific scientific questions with greater precision at lower cost.

**Figure 1.**
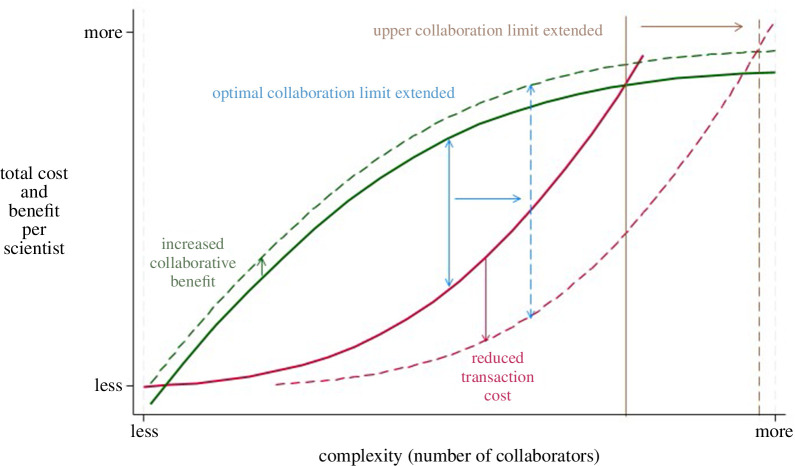
Schematic of extending the collaborative limit by reducing transaction costs and increasing collaborative incentives (adapted from James Buchanan [14] and Richard Cornes and Todd Sandler [15,18]). Key: green solid line: benefit of collaboration according to complexity (size of collaboration). Red solid line: cost of collaboration according to complexity (size of collaboration). Green dashed line: increased benefit of collaboration owing to reduced transaction costs. Red dashed line: reduced cost of collaboration owing to reduced transaction costs. Blue solid and dashed lines: increase in optimal collaboration limit. Brown solid and dashed lines: increased upper collaboration limit.

Figure 1 shows how the benefits of collaboration for knowledge production increase with collaboration (solid green line) owing to the sharing of specialist knowledge and distribution of workload. However, collaboration is not cost free. As the club increases in complexity (size), the costs of establishing and maintaining the collaboration increase (solid red line). This is owing to the growing costs of transacting knowledge and material resources, including complex legal agreements, distributing responsibilities and rewards, ceding peer-esteem indicators to others, etc. At some point, the benefits of knowledge production are outweighed by the costs of transaction, and collaboration stalls. The intersection of benefit and cost curves notionally identifies the upper collaboration limit (solid brown line), whereas the greatest net benefit (benefits–costs) gives the point of optimal collaboration (solid blue line). Although these curves are illustrative and the various cut-points are empirical questions that will vary across disciplines, the essential argument is generally robust.

Club theory suggests the two parallel strategies of increasing incentives (dashed green line) and reducing transaction costs (dashed red line) for extending both the upper collaboration limit (dashed brown line) and the optimal point of collaboration (dashed blue line). Current incentive structures are strongly influenced by what may be described as the competitive dividend. This is not necessarily helpful. In a review of Australian biomedical grant proposals, Herbert *et al*. report that the opportunity cost for preparing new proposals averages at 38 working days, with a 79% failure rate [19]. For the 2966 failed proposals identified in the study, this translated into an aggregate annual salary cost of AU$52 million. Arguably, structures that emphasize the collaborative dividend, resulting in fewer, higher quality proposals, are likely to deliver greater overall benefits at reduced cost.

A key lever to reduce transaction costs is infrastructure. *A priori*, a good science platform will increase the average size of cooperating groups and is likely to yield more scientific products per data asset. UK Biobank provides an example of reducing the search, provenance, legal and many other costs of accessing complex, high-quality longitudinal data [20]. As a result, this platform has become one of the world’s most used biomedical research resources.

These arguments illustrate that collaboration decisions are typically made at the margins, i.e. in terms of private benefit to individual scientists, rather than benefit to the wider scientific community. The extent to which a marginal decision-making calculus will differ from an overall decision-making calculus will vary across scientists, but they are not identical. However, these strategies (increasing collaboration incentives and reducing transaction costs) may be used to narrow the gap between individual and socially optimal decision-making. In this context, funders’ policies are critical to engineering more socially optimal decision-making into the value chain. For example, preferring proposals that use existing infrastructure, and using metrics of third-party data usage to evaluate overall scientific impact.

### 4.2. Making data markets work

In demonstrating the logic of private as well as social benefit in the development of consortia, figure 1 also describes data-sharing behaviour in practice. For biomedical science, a major barrier to scientific advancement is the failure of the data market to provide access to those who can add value. In a survey of 3556 articles from 333 open-access journals, Gabelica *et al*. [21] found that only 7% of corresponding authors responded positively to a data access request, even when their intention to share data was explicitly stated, meaning that in this experiment, the revealed preference of 93% of authors was *not* to share, even if their stated preference was to share. Admittedly, this is a bleak example and can be countered by examples from genetics where only 47% of geneticists reported having a data request denied [22].

If clubs of scientists tend to act to preserve the *internal* (intraclub) shared value of data, it is of interest to consider the mechanisms used. Access to products in data markets is controlled by informal and formal rules that can be thought of as allocating property rights (a right to benefit from a resource). Clearly defined property rights reduce the costs of transacting in markets by providing a secure basis for decision-making and thus incentivize creativity and innovation.

Assigning property rights to data is a social preference. Some preferences, however, are more socially efficient than others in terms of maximizing knowledge creation whilst preserving the interests of the various stakeholders and the incentive structures needed to invest in new scientific knowledge. A rule for socially efficient knowledge creation would require rights to be assigned to those in the best position to use the resource for a desired outcome, such as ‘creating scientific opportunity’, or ‘maximizing the chance of translating discoveries into impacts’. Clearly, such a rule is not typically in place, with de facto rights to further exploit data typically being held by the club of scientists who first created or came to hold ownership of a dataset. In this important sense, for publicly funded science, property rights are unclear and, arguably, socially inefficient [23].

**Figure 2.**
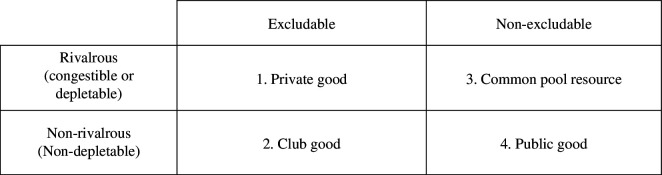
The data market.

Figure 2 shows how property rights may be allocated according to two dimensions that relate to the manner in which data are consumed by users, and by implication the growth of the data market. These are (i) rivalrous consumption, where data access by one party reduces availability to others and (ii) excludable consumption, where one party can exclude others from data access. Rivalrous consumption can be congestible where demand for data access is high, but availability is low (as in a novel high-value dataset), or depletable where the resource is finite (as in a bio-sample collection). Excludability can be on technical, legal, political or ‘professional’ grounds.

Some data are most efficiently organized as a private good when access can be technically, legally and politically, denied (cell 1) [18]. Typically, these data have high novelty, sensitivity and commercial value, and are expensive to create. Under these circumstances, private ownership is crucial to maintaining the production of such data until such time as the costs of creation have been recovered, or reasonable ‘creator rights’ have been exhausted, or the data are replicated more cheaply by new work practices and technologies.

For data that are non-rivalrous but excludable, a ‘club’ (cell 2) is an efficient organizational form. It provides a mechanism for managing data that can cause public harm if mishandled, including threat to the continuing use or existence of the data (or the resource from which data are generated). By excluding non-members, data can be used without congestion and without rivalry within the club, thus protecting their social as well as private value. Club ownership is an efficient solution in such circumstances, with club rights allocated by scientific competence and bona fide purpose.

For other rivalrous resources, such as bio-sample collections, private ownership is unacceptable owing to public funding. These may, on the surface, be considered as common pool resources (cell 3). However, with open access, they are vulnerable to congestion in the form of overuse or suboptimal use, leading to depletion. For these resources, limiting access increases their sustainability and scientific value, giving justification for club ownership, and requiring perhaps stricter membership conditions.

We also note, however, that some data are both non-rivalrous and non-excludable (cell 4). These are pure public goods in terms of consumption characteristics and are most efficiently placed in the public domain, i.e. with rights given to all. Some data become like this over time, where the value added to data by multiple entrepreneurial scientists and citizens does not devalue the data for others. Data that are technically excludable but unlikely to be congested by open use are typically inefficiently organized by either scientific clubs or privately by the data’s creator. As a general strategy, restricting property rights to such data is a low-growth, low-innovation path. In the 1980s, the UK Ordnance Survey decided to charge premium sums to access their spatial databases. The USA took the opposite approach, making a raft of location-based databases free to all users who would add value. Subsequent growth in the US geographical data science community was several orders of magnitude greater than in the UK. Non-congestible scientific data that are technically excludable are best not made exclusive. Changes in data access rights, platform management protocols, grant conditions and so on can be designed to remove exclusive access after the period during which exclusive access is appropriate. Within science communities, there is a lack of clarity in switching rights from exclusive to the public domain. This arises because funders with a fiduciary responsibility to maximize social benefit from research funds tend to give away too much of their right to grant bidders, who capture it for their own benefit.

These distinctions allow the application of non-arbitrary and transparent rules of access. Although assigning property rights to those best-placed to achieve the desired outcome are user-community judgements, the principles upon which these judgements rest can be made more explicit. For example, while clubs may be the natural order for much biomedical science, eligibility for club membership depends on judgements of who can add value and at what cost. An example of how making property rights explicit supports growth comes from UK Biobank. Here, property rights were addressed through a single standard data controllership agreement; leading to an approval rate of 99% (UK Biobank: private correspondence with Naomi Allen, 2023). Such platforms effectively manage responsible access on behalf of multiple contributors, achieving economies of scale and protecting against data ‘congestion’. Platforms can be thought of as shifting data along the continuum from being a restrictive club good, to being a less restrictive club good via a larger club and via federations of clubs, to achieve greater social benefit. Where platform approval equals approximately 100% of applications, it has effectively converted an inefficient club good into an efficient public good.

### 4.3. Constrained user-led innovation

The self-organization of scientists into clubs of different size and function evolves over time. Never at equilibrium, clubs will vary in size, longevity and configuration. As we have discussed, these trajectories will be determined by the dynamics of benefits and costs. For those concerned with building high-quality research environments, a strategic question is: can we grow the science base through manipulating the organization of complex knowledge production?

Top-down solutions typically lack agility and responsiveness in the face of changing needs, while spontaneous, decentralized, user-led solutions are hallmarks of an innovative and open research culture. However, uncoordinated and competitive user-led solutions can also generate perverse outcomes. These include multiple idiosyncratic data models, poorly annotated data, poorly documented metadata, duplicative non-standard data processing pipelines and wasteful intergroup competition. All such transactional frictions contribute to increased production and transaction costs through extended research cycles and non-reproducible analyses. In this way, over-decentralization makes accessing the data market more difficult.

To address this dilemma, it is helpful to understand that successful decentralized systems of exchange require institutions that underpin efficient collective behaviour. From informal rules that operate within individual laboratories, to legal obligations regarding data access, agreements emerge that constrain some activities in order to promote others. In market-driven sectors, this leads to industry-organized standards. These institutions provide the structures that govern the activity of scientific clubs. Where these institutions do not operate, activity within and between clubs is uncoordinated and duplicative, and efficient solutions to emerging challenges are slow to develop. Counterintuitively perhaps, in order to promote diversity and autonomy, deliver more ‘jam for today’ and be responsive to change, a level of coordination is required.

In a data-driven economy, institutions governing data standards deserve close attention. Data standards can increase the dynamism of club activity by increasing the marginal benefits of club membership. This is the underlying theory behind the development of curated research repositories. Such standards exist elsewhere in science with different organizations developing them for their own communication, e.g. for computing support for collaboration (CSCW [24]) physics (NIST [25]), astrophysics (IVOA [26]) and molecular biology (ELIXIR [27], GA4GH [28]). In biomedicine, one example is reference SNP cluster ID (rs) numbers [29]. By establishing rules around how (and how not) to annotate genetic data, confidence in the provenance of data is increased, transaction costs of data access are lowered and the pooling of property rights to support rapid publication is incentivized. The introduction of rs numbers has been transformative.

Curated reference repositories also have a role for cohort studies. Cohort studies are a critical component of biomedical science’s armamentarium. The growing importance of cohorts is demonstrated in that, for dementia alone, the number of cohort-based publications per year increases monotonically, currently exceeding 2000 per year [30]. Each cohort tends to use a bespoke data model and governance structure that has evolved over time; typically, according to the scientific priorities and resource constraints of the study. This incurs substantial knowledge production costs and leads to significant transaction costs for third-party researchers. A recent study comparing data preparation times for 25 variables in two cohorts found that using ‘bespoke’ cohort-designed data models required 5–6 h per cohort, while using a standard data model reduced this time to 30 min per cohort [31]. The global cost to science of maintaining this inefficiency when an alternative strategy is available is substantial.

Although the ‘in-principle’ value of curating cohort data to a defined standard is widely accepted, the biomedical data economy is heavily incentivized to deliver immediate insights, rather than enable more insights to be made. For example, data are typically curated for internal consumption using bespoke structures and conventions, and minimal documentation. Such data are not readily accessible by third-party researchers. In figure 1, this is consistent with the vertical blue line (privately optimal for club members) rather than the vertical brown line (socially optimal). The rise of multi-cohort data management platforms demonstrates the value of collective data management and provides an opportunity for a more strategic approach to data. The argument is that funders can collectively incentivize the science community to engage in win–win collaboration by establishing efficient institutions that expand productive collaboration. An example of this is Health Data Research UK [32], which addresses these issues primarily for administrative health data.

Arguably, non-proprietary research funders exist to support collective scientific goals and activity, and have a role in supporting the science community to develop efficient institutions; not least because these make science attractive to potential scientists, avoid wasting resources and grow the national innovation base. However, funders tend to eschew this subtle but critical role, as it can be interpreted as an imposition on academic freedoms. An alternative perspective is that funders have a responsibility to work with scientists to support the development of mature institutions that provide an efficient science data ecosystem. The aforementioned development of rs numbers is illustrative. The rs number solution was generated by the scientific community in response to the need for greater reproducibility. But, the emergence of rs as a standard was accelerated by the leadership of stakeholders with critical mass. It was the coordinated action of two major stakeholders (National Center for Biotechnology Information [33] and National Human Genome Research Institute [34]) that realized the ambition. Without this leadership, it is likely that rs numbers would have been just one of a number of (equally useful) solutions; none of which achieving widespread adoption.

### 4.4. Trust-by-design

Trust is the implicit operating principle underlying human collective activity. It is fundamental to the formation and cohesion of scientific clubs. Trust simplifies otherwise complex and unpredictable environments by identifying points of certainty around which to organize, and agreeing on a culture where key uncertainties are removed on the basis of mutual agreement, respect and ethical codes. By facilitating better prediction of the likely reciprocal behaviour of others in sharing costs and benefits, trust fosters collaboration through the efficient assignment of informal and formal property rights and benefits, which are at the core of club membership decisions. Trust is also foundational to the provenance of data and technologies. As datasets grow in size, complexity and sensitivity, confidence in the provenance chain becomes increasingly central to the viability of the entire science economy.

Informal trust-based solutions work well for bilateral collaboration and can work for small communities. However, this limits club size and the scope of question it can address. Emerging research questions frequently require multi-lateral collaboration involving large numbers of diverse stakeholders, and for these, time-tested, less-formal solutions are inadequate. Multiple actors with competing interests involving various data sources generate complexity leading to potentially prohibitive coordination costs. An alternative strategy for managing multi-lateral collaboration is trust-by-design. Here, legal, privacy, security and scientific requirements are embedded within technical and organizational workflows that are explicit, transparent and fully auditable. These workflows can be user-designed or service-provider-designed according to need. This enables systematic streamlining, standardization and automation. Although designed to service multi-lateral collaboration, embedded workflows have utility for collaboration in general. Trust-by-design solutions provide the information necessary for accurate and rapid judgements of trustworthiness and scientific value. It involves a shift from trust in a person or a group to trust in a system.

Trust-by-design offers unprecedented scientific agility. Clubs can form at reduced cost and time. Existing clubs can easily coalesce to form ‘task and finish’ super-clubs to address specific scientific challenges, and it enables the efficient management of what may be called mega-clubs. Dementias Platform UK is an example of an emerging mega-club enabling more than 1000 third-party analysts in 48 countries to analyse survey, imaging, genomics and device data at low or no cost [35]. AMP-AD [36] in the USA and the globally reaching AD Workbench [37] are other emerging mega-clubs. For prospective data collection, mega-club models are exemplified by several large population studies that have been established as scientific resources, including All of Us [38] and Our Future Health [39]. An innovative approach to trust-by-design, using swarm computing, is the use of blockchain to deliver transparent and permanent governance for decentralized peer-to-peer networking [40]. This dispenses with the need for a central coordination. With the increasing complexity and sensitivity of emerging datasets, trust-by-design is likely to prove the only scientifically efficient and socially acceptable solution for high-quality research environments. Distributed-ledger technology such as blockchain has the effect of removing barriers to trust by replacing central coordination with distributed coordination. This is likely to increase the size and social benefit of data-sharing collaborations.

### 4.5. On motivation

Our brief survey of abstracts shows that to many scientists it is important that their club membership contributes some form of scientific and social benefit. It is worth considering what this does and does not mean for research environments.

The intellectual underpinning of science comprises observation, causal inference and application. As scientists, we are familiar with the systematic observation of phenomena and consider this delivers objectivity. We are also familiar with deriving insight by applying causal inference to observation. Both activities are considered intrinsically aesthetically satisfying. Furthermore, their utility lies in being untrammelled by extrinsic judgements of social benefit.

For application, however, the situation is reversed. Austrian philosopher and logician, Wittgenstein, observed that if we compiled an exhaustive book of all the observations in the universe, and the insights derived, it would not contain a single objective value statement [41], i.e. not provide a basis for objectively evaluating social benefit. It appears that as scientists, we are compelled to make judgements of value that cannot ultimately be derived from observation or inference. Responses to this predicament vary and are contingent on the state of knowledge. Nevertheless, at the root of every value chain there lies what may be described as an ethical judgement. This is brought into sharp relief with advances in machine learning: the more complex the algorithm, the more salient the need to judge its ethical value. For the operation of scientific clubs, confusing aesthetic and ethical judgements is unhelpful. For example, the desire for inclusive science has both aesthetic benefit (more informative data) and ethical benefit (reduced inequality). They are closely entwined but distinct, involving different and sometimes conflicting priorities that need to be clearly understood for optimal decision-making.

In relation to research environments, the systems and practices that address both aesthetic and ethical concerns are likely to be most attractive. The more deeply we understand the aesthetic and ethical implication of our research, the better positioned we are to design research environments that command public respect and support, and that motivate individual scientists and clubs thereof, to invest in the ‘hard miles’ that rigorous science requires. In the end, these considerations are important for the social reproduction of science within society. But perhaps, for the consideration of research funders and others in a position to lead a change of culture, the motivational calculus more powerfully begins with the ethical.

## 5. Discussion: towards high-quality research environments

Research environments, almost by definition, increase in complexity with each step in scientific specialization. The question addressed here is whether there are characteristics of research environments that are reasonably common across aligned disciplines, and which provide a framework for guiding the development of specific solutions for particular communities. The problem is configured as how to facilitate growth in a data-driven economy. A literature review identified five salient characteristics of research environments comprising collaboration, data access, innovation, trust and motivation. These were explicated using economic club theory.

### 5.1. Strengths

**Figure 3.**
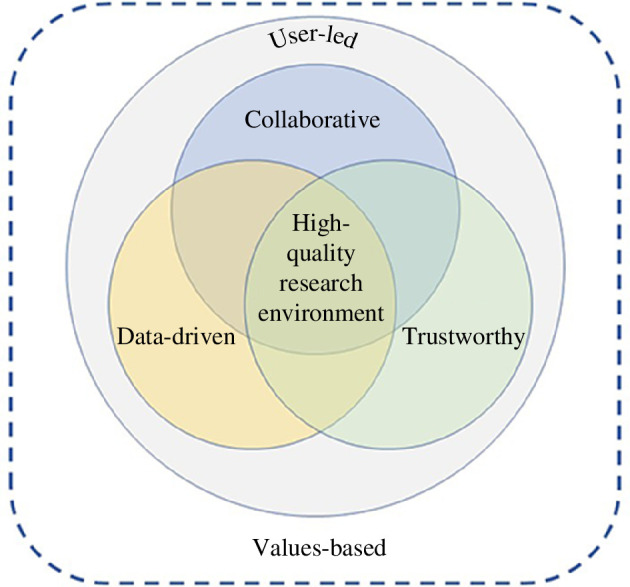
A model for high-quality research environments.

Strengths of the study include providing a clearly defined model for evaluating biomedical research environments. At one level, the model components are not individually surprising; they are pervasive to every scientist’s experience. However, to consider their distinctive impacts on decision-making, and how this contributes to the quality of a research environment is informative. Providing a model for the impacts of social versus private benefits on collaboration, the allocation of property rights on data access, developing efficient institutions, creating trust and being explicit about our value base, provides a guide for more productive and satisfying laboratory behaviour, consortium development and strategic funding-policy decisions. Figure 3 is a schematic of this model showing how a value-based and user-led innovation provides the context and engine underlying collaboration, data access and trustworthiness. While the model is primarily applied to biomedical environments, it may also have wider relevance.

An implicit tension between competition and collaboration underlies the argument and shapes any scientific data economy. Competition between scientists and a judicious degree of private property rights provide a necessary incentive for the investment of personal energy, resources and funding into worthwhile projects, thus sustaining the quality of science. Competition between funders, in the sense of complementary agendas, also helps maintain the quality of science, particularly in the originality of research. However, there is a substantial collaborative dividend available for synergizing and not duplicating effort. Remove competition from either supply or demand sides, and the quality of funding calls and bids declines. However, to improve overall benefit from limited resources, aspects of competition need moderating.

To convey the argument, we have characterized this as a problem of club formation, since scientists, by and large, behave as though scientific data are club goods. One distinction of a good that is best organized in clubs is that the efficient quantity and quality of the good (research) is determined simultaneously with club size. This clearly happens routinely as scientists invite/join collaboration based on the likely outputs of a particular configuration. Our argument is that while this type of market is more productive than a market of individually competing Principal Investigators without clubs, it nevertheless undersupplies science. In elaborating the argument, we point to platform technologies and collaborative incentives as market-corrective mechanisms that are likely to lower the transaction costs that hinder collaboration.

We argue that providing an efficient ecosystem in which these negotiations can take place requires the development of mature institutions around practice. These institutions are not restrictive, but supportive of decentralized and spontaneous decision-making, and provide the conceptual framework around which automated procedures that serve the interest of all stakeholders can be developed. The example given from swarm computing is germane. Here, the use of blockchain technology enables autonomy and diversity, but only because it is used as a trustworthy institution. Funders have a key leadership role in coordinating these efforts.

Finally, we focus on the value base that underpins the science ecosystem; understanding the distinction between aesthetic and ethical judgement. We argue that promoting both motivates high-quality science by providing a framework for personal motivation, the efficient formation of clubs to address specific challenges and the coalescence of clubs into ‘mega-clubs’ for addressing multiple and specific scientific challenges.

Although our focus has been on biomedical science, this is an exemplar, and the model has application to data-driven science more widely. This applies particularly to the Global South, where there is opportunity to address foundational structures prospectively rather than inherit some of the unhelpful constraints of current practice.

### 5.2. Limitations

A limitation of this paper is that it does not provide specific solutions for particular communities. However, specific solutions have a ‘shelf-life’ that reflects current scientific needs and available resources. Here, we are concerned with the structures that constrain decision-making and so constrain the development of solutions. The extent to which these five characteristics are transient or persistent will be an interesting natural experiment. We suspect they are fundamental to the creation of complex knowledge, and so are likely to be enduring.

The analysis is limited in being based on a constrained systematic literature review. A more detailed search would have revealed a larger and richer tapestry. But it would probably also have been more focused on discipline-specific solutions. Whether a more detailed search would be more informative at the structural level, however, is moot. In identifying salient characteristics of research environments, the search strategy achieved its purpose.

A further limitation is the use of theoretically driven cost–benefit curves (figure 1). In the absence of relevant data, this is unavoidable. While the underlying theory is informative, the curves are illustrative of general principles. The actual shape of these curves and optimal levels of complexity will vary according to technology, research question and research group ethos. The curves perform as a qualitative logic for our arguments about size, quantity, quality and private and social productivity of scientific collaborations.

The definition of research environment in terms of a data economy is not comprehensive and other perspectives will add understanding. The focus on the biomedical sciences does not do justice to developments in the data environments of the physical sciences. Arguably, however, the sensitivity of biomedical data brings governance and social responsibility constraints that urgently need addressing.

The definition of ‘club’ used here, that of a generic research consortium, does not do justice to the richness of the concept. Clubs may form around any use of a public good. For making generalizable points around biomedical research environments, however, the level of specificity is reasonable.

### 5.3. Next steps

The model requires developing and empirical testing as cost–benefit curves will vary according to context. Identifying the determinants and parameters of this variation for specific scientific communities would enable the model to be used for strategic planning with greater confidence.

Also, the model can act as a stimulus, encouraging scientific communities to develop standards and frameworks that encourage efficient science. For example, developing consensus around phenotype classifications, and data management protocols.

The model, while not complete, provides directions of travel for the development of scientific communities. In particular, it provides a conceptual framework for rebalancing tensions between the competitive independent scientist and the collaborative scientific community, for mutual benefit. In this respect, funders have a strategic role. The collective introduction of governance expectations and supporting metrics to produce an efficient data market, would benefit all stakeholders; increasing their scientific output.

## 6. Conclusions

More optimally configured collaborative scientific groupings have the potential to raise the dividend of science funding, increase global competitiveness, raise the speed of advancement in science and civilization, increase scientific opportunity, bring more people into science and improve career satisfaction. While the issues raised are largely structural and unresolvable by individual research groups, they can be addressed by research communities and funders. In this context, our framework provides a direction of travel and a basis for debate. In so doing, it attempts to help science communities rise above self-interest and provide a sense of common purpose that is characteristic of science at its best. The challenge is not trivial but the prize is great.

## Data Availability

The results of the literature review are included as supplementary material [42].
